# A Selective HDAC 1/2 Inhibitor Modulates Chromatin and Gene Expression in Brain and Alters Mouse Behavior in Two Mood-Related Tests

**DOI:** 10.1371/journal.pone.0071323

**Published:** 2013-08-14

**Authors:** Frederick A. Schroeder, Michael C. Lewis, Daniel M. Fass, Florence F. Wagner, Yan-Ling Zhang, Krista M. Hennig, Jennifer Gale, Wen-Ning Zhao, Surya Reis, Douglas D. Barker, Erin Berry-Scott, Sung Won Kim, Elizabeth L. Clore, Jacob M. Hooker, Edward B. Holson, Stephen J. Haggarty, Tracey L. Petryshen

**Affiliations:** 1 Center for Human Genetic Research, Massachusetts General Hospital, Boston, Massachusetts, United States of America; 2 Department of Psychiatry, Harvard Medical School and Massachusetts General Hospital, Boston, Massachusetts, United States of America; 3 Department of Neurology, Harvard Medical School and Massachusetts General Hospital, Boston, Massachusetts, United States of America; 4 Department of Radiology, Harvard Medical School and Massachusetts General Hospital, Boston, Massachusetts, United States of America; 5 Athinoula A. Martinos Center for Biomedical Imaging, Massachusetts General Hospital, Department of Radiology, Harvard Medical School, Charlestown, Massachusetts, United States of America; 6 Stanley Center for Psychiatric Research, Broad Institute of Harvard and MIT, Cambridge, Massachusetts, United States of America; 7 Medical Department, Brookhaven National Laboratory, Upton, New York, United States of America; Radboud University, The Netherlands

## Abstract

Psychiatric diseases, including schizophrenia, bipolar disorder and major depression, are projected to lead global disease burden within the next decade. Pharmacotherapy, the primary – albeit often ineffective – treatment method, has remained largely unchanged over the past 50 years, highlighting the need for novel target discovery and improved mechanism-based treatments. Here, we examined in wild type mice the impact of chronic, systemic treatment with Compound 60 (Cpd-60), a slow-binding, benzamide-based inhibitor of the class I histone deacetylase (HDAC) family members, HDAC1 and HDAC2, in mood-related behavioral assays responsive to clinically effective drugs. Cpd-60 treatment for one week was associated with attenuated locomotor activity following acute amphetamine challenge. Further, treated mice demonstrated decreased immobility in the forced swim test. These changes are consistent with established effects of clinical mood stabilizers and antidepressants, respectively. Whole-genome expression profiling of specific brain regions (prefrontal cortex, nucleus accumbens, hippocampus) from mice treated with Cpd-60 identified gene expression changes, including a small subset of transcripts that significantly overlapped those previously reported in lithium-treated mice. HDAC inhibition in brain was confirmed by increased histone acetylation both globally and, using chromatin immunoprecipitation, at the promoter regions of upregulated transcripts, a finding consistent with *in vivo* engagement of HDAC targets. In contrast, treatment with suberoylanilide hydroxamic acid (SAHA), a non-selective fast-binding, hydroxamic acid HDAC 1/2/3/6 inhibitor, was sufficient to increase histone acetylation in brain, but did not alter mood-related behaviors and had dissimilar transcriptional regulatory effects compared to Cpd-60. These results provide evidence that selective inhibition of HDAC1 and HDAC2 in brain may provide an epigenetic-based target for developing improved treatments for mood disorders and other brain disorders with altered chromatin-mediated neuroplasticity.

## Introduction

Epigenetic mechanisms involving chromatin-modifying enzymes and remodeling factors are increasingly implicated in the pathophysiology of mood (affective) disorders including depression and bipolar disorder, as well as in other psychiatric diseases such as schizophrenia [1]. Neuroplasticity – the capacity for changes in brain function – is relevant to understanding both disease states and effective treatment mechanisms. These changes involve dynamic modulation of chromatin– DNA packaged around octameric cores of histone proteins H2A, H2B, H3 and H4 - which is subject to diverse post-translational modifications. Acetylation of histone amino-terminal tails is associated with an open chromatin structure that facilitates the binding of transcriptional activating protein complexes that modulate gene expression [2] and alter neural circuit function. Histone deacetylase (HDAC) enzymes, including subtypes comprising class I (HDAC1, 2, 3 and 8) and class II (HDAC 4–7, 9 and 10), control the deacetylation of histone and non-histone proteins. These enzymes are therefore important mediators in epigenetic regulation of gene expression that may contribute to mechanisms underlying psychopathology and treatment.

Recent findings indicate that the activity of specific class I and II HDAC enzymes may be altered in psychiatric disease and may play a role in effective clinical treatments.

Postmortem studies have revealed altered mRNA and protein expression of HDAC1, 2 and 5 among patients with major depressive disorder, schizophrenia and bipolar disorder [3]–[5]. Valproate, a drug widely used in bipolar disorder treatment, functions in part as an inhibitor of all class I HDACs [6], [7]. Moreover, lithium therapy, a mainstay bipolar disorder treatment and antidepressant adjunct, as well as the typical antipsychotic, haloperidol, were shown to increase histone acetylation in cellular and animal models [8]–[11]. Further, HDAC2 was recently demonstrated to be a key regulator of atypical antipsychotic response [12]. Thus, investigating altered histone acetylation in the context of mood and psychotic disorders may provide insight toward critical factors regulating plasticity as well as novel therapeutic targets based on epigenetic mechanisms.

Animal model research further supports the link between HDAC activity and mood disorders. Electroconvulsive therapy, used in treatment-resistant depression, was shown to alter histone H3 and H4 acetylation at the promoter regions of actively transcribed genes in rat hippocampus [13]. Additional rodent behavioral data demonstrate antidepressant-like effects of the class I HDAC inhibitor, sodium butyrate [14], the HDAC1/2/3 inhibitor, MS-275 [3], as well as reduced psychostimulant-induced hyperactivity by valproate and sodium butyrate [15], [16]. However, these reports used weak inhibitors with low selectivity for different class I HDAC subtypes that may engage non-HDAC targets at high physiological concentrations (millimolar range). Thus, the class I HDAC subtypes critical to the observed effects remain unclear.

To further investigate the mechanism of HDAC inhibition in the underpinnings and treatment of mood disorders, we identified from the literature Cpd-60 (Compound 19, also published as Compound 60), a benzamide-based, subclass selective inhibitor of HDAC1 and HDAC2 [17], [18]. Cpd-60 has structural features distinct from previously studied compounds that make it an excellent probe compound. We demonstrate here, for the first time, that chronic treatment of mice with Cpd-60 results in substantial effects in two behavioral tests with predictive validity for mood stabilizer and antidepressant medications. Cpd-60 treatment was associated with significant gene expression changes in prefrontal cortex (PFC), nucleus accumbens (NAc) and hippocampus (HIP), brain regions involved in the regulation of mood [19], [20], through an HDAC inhibition-mediated mechanism evidenced by increased histone acetylation at gene promoter regions. Interestingly, a small subset of gene expression changes induced by Cpd-60 significantly overlap with those induced by lithium, suggesting common mechanistic elements that may play a role in altering behavior. Together, this study demonstrates that selective inhibition of HDAC1 and HDAC2 in mice modulates transcription in mood circuits and alters relevant behaviors, and may be a viable mechanism for the development of clinical mood disorder treatments.

## Materials and Methods

### Chemical Synthesis

Cpd-60 and SAHA were synthesized according to published protocols [18], [21], [22]. All compounds were greater than 95% purity and stored at −20°C as dry powders prior to use.

### Animals

Male 11 wk old C57BL/6 mice were utilized for pharmacokinetic, behavioral and biochemical analyses. One female baboon (*Papio Anubis*) was used to determine brain uptake and pharmacokinetics of Cpd-60. *Ethics Statement:* All animal work was approved conduced under strict accordance to the ethical standards set by the Massachusetts Institute of Technology Institutional Animal Care and Use Committee (IACUC) and the Committee on Animal Care (mouse experiments, internal protocol #0410-03-013) and by the Brookhaven National Laboratory IACUC (baboon experiment, protocol #102). Non-human primate housing conditions and feeding regimens were coordinated by the professional investigative staff at the Brookhaven Laboratory Animal Facility which included social housing in cages appropriate for the physical and behavioral health of the individual animal. Animals were fed a 3x per day with additional nutritional supplements provided as prescribed by the attending veterinarian. Environmental enrichment included audio, video and tactile elements (e.g. listening to the radio, watching television, playing with toys and human interaction) and were provided on a daily basis to promote psychological well-being. All procedures were performed without compromising animal welfare and all efforts were made to minimize suffering including adequate use of anesthesia (ketamine, isoflurane) in the baboon imaging experiment. The baboon was not sacrificed following the study and further effort was taken to minimize suffering by allowing an interval of at least one month between subsequent imaging studies in the same animal.

### Pharmacokinetic Profile Determination

Mice were treated (i.p.) with Cpd-60 (45 mg/kg) or SAHA (25 mg/kg) in vehicle (10% DMSO, 45% PEG400, 45% saline) and blood collected by retro-orbital puncture into heparinized tubes at pre-treatment, and 0.083, 0.25, 0.5, 1, 2, 4, 6, 8 and 24 hr post-treatment (*n = *3 mice/group), followed by immediate sacrifice and brain harvest. Plasma, brain samples, and dose formulations, were analyzed as previously described [23] using high performance liquid chromatography/mass spectrometry. Data acquisition and control system were created using Analyst 1.4 software (ABI Inc, Canada).

### CNS Target Binding Assays

Cpd-60 was submitted to a panel of 80 binding assays for common transmembrane and soluble receptors, ion channels and monoamine transporters in the central nervous system (CNS) (High-Throughput Profile P-3, Cerep, France). Cpd-60 (10 µM) was assayed in duplicate concurrently with an assay-specific reference compound (Table S1).

### Biochemical Assays

HDAC activity was measured *in vitro* using recombinant human HDACs 1-9 (BPS Bioscience) using the Caliper EZ reader II system. *HDAC inhibition assays:* Purified HDACs were incubated with a FAM-labeled fluorescent substrate and test compound at room temperature for 60 min or, for HDAC 1-3, 180 min to control for effects of slow-binding inhibitors on HDAC activity. Fluorescence intensity of electrophoretically separated substrate and product was measured and the percent inhibition plotted against compound concentration. IC_50_ values were determined by curve fitting with Origin 8.0 software [24]. *Binding kinetics:* Binding kinetics of Cpd-60 and SAHA with HDACs 1, 2, and 3 were evaluated by progression curves in inhibition and dilution experiments as previously described [25].

### Mouse Primary Neuronal Histone Acetylation Assays

Mouse primary neurons cultured 13 days *in vitro* were treated with HDAC inhibitors for 24 hr, fixed with 4% formaldehyde, and stained with an anti-acetylated H4 lysine 12 antibody and an Alexa-488 conjugated secondary antibody, with nuclei identified using a Hoechst stain. Cells with histone acetylation signals above an intensity threshold of >99.5% were scored as “bright green cells” and expressed as a percentage normalized to DMSO controls. EC_50_ values were determined from curve fitting using GraphPad Prism v5 software (GraphPad Software, Inc., USA).

### Pharmacological Treatments

Cpd-60 (45 mg/kg, 7.5 mL/kg, i.p.) and SAHA (25 mg/kg, 5 mL/kg, i.p.) were prepared fresh for daily injection in vehicle (10% DMSO, 45% PEG-400, 45% saline). D-amphetamine (Sigma-Aldrich) was prepared in saline and administered via i.p. injection (3.5 mg/kg, 5 mL/kg).

### Behavioral Procedures

Compounds were administered between 9 am and 1 pm for 7 days prior to the start of behavioral testing, and after completion of each daily session during behavioral testing. Testing was performed 18–24 hr after the previous treatment to avoid transient effects. Amphetamine-induced hyperlocomotion (AIH) was performed as previously described [26] on days 7–9 of treatment with activity measured before and after amphetamine challenge (AccuScan Instruments, Inc.). Forced-swim test (FST) behavior was performed as previously described [26] on day 10 of treatment and total time spent immobile during a 6 min session was scored automatically (Ethovision; Noldus). Treatment effects were analyzed by one way analysis of variance (ANOVA) with *post hoc* analysis using Least Squared Differences test (SPSS v18.0 IBM).

### Brain Tissue Collection

Behaviorally naïve mice were treated daily (i.p.) with Cpd-60 (45 mg/kg), SAHA (25 mg/kg) or vehicle. One hour after the last of 10 treatments, harvested brains were snap frozen and stored at −80°C until use. Independent sets of treated brains were used for western blotting, transcriptional analysis, and chromatin immunoprecipitation, as detailed below.

### Western Blotting

Frozen mouse brains (*n* = 6/treatment group) were rapidly dissected at 4°C to isolate frontal cortex (including PFC), ventral striatum (including NAc), and HIP for protein extraction. Human Embryonic Kidney (HEK) 293 cells were treated with 20 µM Cpd-60, SAHA or DMSO for 24 hr ‘constant’ treatment, followed by media change and 6 hr incubation (‘washout’), and cells collected for protein extraction. Solutions for protein extraction were supplemented with 5 mM sodium butyrate to suppress residual HDAC activity. Western blotting was performed using standard protocols and commercially available antibodies (Millipore) raised against acetylated histone H2B (07-373), H3K9 (07-352), and H4K12 (04-119) with normalization to total levels of histone H3 (07-690) or histone H4 (04-858). Densitometric quantification was performed using Image J software (NIH) and statistical comparison to vehicle-treated controls by two-tailed t-test.

### RNA Isolation and Transcriptional Analysis

Frozen mouse brains (*n* = 6/treatment group) were rapidly dissected at 4°C to isolate medial prefrontal cortex (PFC), nucleus accumbens (NAc) and hippocampus (HIP) for mRNA extraction. Whole genome transcript profiling was performed using the Illumina MouseWG-6 Expression BeadChip with subsequent validation by quantitative real-time PCR (qPCR) and is available online at the NCBI Gene Expression Omnibus repository (GSE47452).

### Chromatin Immunoprecipitation

Native chromatin immunoprecipitation (ChIP) was performed as previously described with minor modifications [27]. Frozen mouse brains treated with Cpd-60 or vehicle (*n = *4/group) were rapidly dissected at 4°C and NAc isolated. Nucleosomal chromatin (‘Input’) was prepared by digesting native (unfixed) chromatin with micrococcal nuclease. 100 µL of the ChIP product was incubated overnight with anti-histone H4K12ac antibody and precipitated using agarose beads (Santa Cruz #SC-2003). Purified, immunoprecipitated DNA was applied to qPCR using primers (Table S2) designed using NCBI PRIMER-BLAST targeting regions proximal to gene transcription start sites (TSS). Resulting DNA amplification curves were used to calculate the ratio of immunoprecipitated DNA from ChIP to Input with statistical significance determined by two-tailed t-test of Cpd-60 or SAHA compared to vehicle.

## Results

### Characterization of Cpd-60 as a Brain-penetrant, Selective Inhibitor of HDAC1 and HDAC2

**Figure 1:**
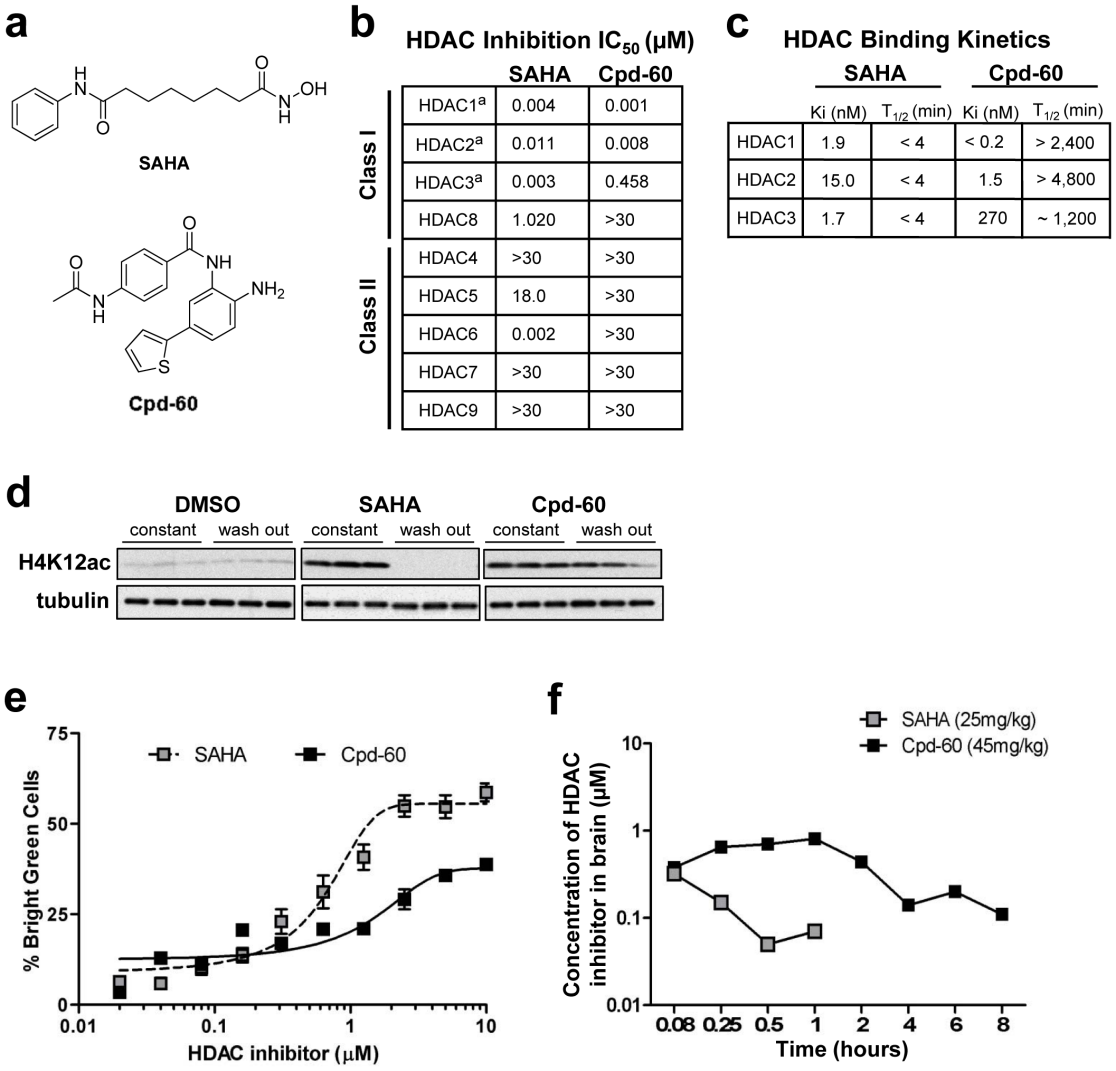
*In vitro* and *in vivo* characterization of two structural classes of HDAC inhibitors. (**a**) Chemical structure of SAHA and Cpd-60. (**b**) *In vitro* IC_50_ (µM) for HDAC 1-9 by SAHA and Cpd-60 using recombinant human HDAC enzymes and HDAC class-specific substrates. Inhibitor and substrate were incubated for 60 min (HDAC4-9) or 180 min (HDAC1-3)^a^ to control for HDAC1-3 inhibition by slow-binding test compounds. (**c**) *In vitro* binding affinity (K_i_) and kinetics (half-life ‘T_1/2′_ in minutes) for HDAC 1, 2 and 3 incubated with SAHA or Cpd-60 (10 µM). (**d**) H4K12 acetylation levels in HEK293 cells following 24 hr ‘constant’ exposure to DMSO, SAHA (20 µM) or Cpd-60 (20 µM) and 6 hr after drug ‘washout’ (media change) with tubulin loading control. (**e**) Dose response plots for induction of histone H4K12 acetylation in cultured primary mouse neuronal cells by SAHA or Cpd-60 for 24 hr. Cells with histone acetylation signals above an intensity threshold of >99.5% (“bright green cells”) are plotted as a percentage normalized to DMSO control. EC_50_ values for H4K12 acetylation were 0.60 µM and 72 µM for SAHA and Cpd-60, respectively. (**f**) *In vivo* mouse brain pharmacokinetics following acute systemic administration of SAHA (25 mg/kg, i.p.) or Cpd-60 (45 mg/kg, i.p.).

In order to assess the utility of Cpd-60 (Fig. 1a) for *in vivo* studies, we initially determined its target selectivity and potency, binding kinetics, activity in cultured mouse neurons and brain penetrance. For comparison, we also assessed these parameters for SAHA (Fig. 1a), a non-selective inhibitor of class I and class II HDAC subtypes that has been demonstrated in mice to enhance cognition following chronic systemic treatment [28] and improve depression-related behaviors when directly infused into brain [3].

Using a fluorometric biochemical assay (Fig. 1b.), we confirmed the selective inhibition by Cpd-60 of HDAC1 and HDAC2 (IC_50_ = 1 and 8 nM) with 50–400 fold selectivity over class I HDAC3 (IC_50_ = 458 nM), and no appreciable inhibition of HDAC8 or of the class II HDACs (IC_50_>30 µM). Additional biochemical assays revealed high-affinity (K_i_ = 0.2–1.5 nM) and slow-on/slow-off binding kinetics of Cpd-60 to HDAC1 and HDAC2 with half-lives (T_1/2_) of 40- and 80-hr (2400–4800 min; Fig 1c). Cpd-60 had lower affinity (K_i_ = 270 nM) and engagement of HDAC3 (T_1/2_ = 20 hr Fig.1c). In comparison, SAHA exhibited potent inhibition of HDAC1, 2, 3 and 6 (IC_50_ = 2–11 nM; Fig. 1b), similar to previously published results [6], and fast-on/fast-off binding kinetics (T_1/2_<4 min for HDAC1–3; Fig. 1c).

A subsequent counter screen against 80 common CNS targets including receptors, channels and transporters (Table S1), identified a clean profile for Cpd-60 (10 µM), with no significant binding to any of the targets (50% inhibition of control binding). These data suggest that biological effects of Cpd-60 are likely due to its inhibitor activity towards HDAC1 and HDAC2, and are not due to off-target effects.

To determine whether the binding kinetics of Cpd-60 and SAHA correlated with changes in cellular HDAC activity over time, we examined the acetylation of histone H4 at lysine 12 (H4K12ac) by western blot in HEK293 cells exposed to Cpd-60 or SAHA. Treatment with either inhibitor (20 µM for 24 hr) elevated H4K12ac levels (Fig. 1d, ‘constant’), however 6 hr after media change (Fig. 1d, ‘washout’), only Cpd-60-treated cells showed persistent increases in H4K12ac, indicating lasting HDAC binding and functional inhibition. To verify neuronal HDAC inhibitory activity of Cpd-60 and SAHA, we measured histone acetylation in cultured primary mouse forebrain neurons using an immunofluorescence-based, laser-scanning cytometry assay. Treatment with Cpd-60 or SAHA (20 µM) for 24 hr induced dose-dependent acetylation of H4K12ac (Fig. 1e) with EC_50_ values of 7.2 µM and 0.6 µM for Cpd-60 and SAHA, respectively, demonstrating robust inhibition of HDACs in cultured neurons.

Brain pharmacokinetic analyses (Fig. 1f) using doses corresponding to subsequent behavior and transcript expression studies determined that Cpd-60 had sustained brain exposure (T_1/2_ = 6.44 hr) compared to SAHA (T_1/2_ = 0.44 hr). The maximum concentration (C_max_) of Cpd-60 was 0.83 µM, far exceeding the *in vitro* IC_50_ for HDAC1 and HDAC2 (0.001 and 0.008 µM) but not HDAC3 (0.4 µM), suggesting robust inhibition of HDAC1 and 2. Preliminary positron emission tomography data following intravenous treatment of carbon-11 labeled Cpd-60 in baboon, collected as previously described [29], were consistent with rodent data and confirmed that Cpd-60 reaches the brain, albeit at low levels relative to plasma (S.W.K., J.M.H. unpublished data).

Overall, Cpd-60 was confirmed to be brain penetrant with selectivity and slow-on/slow-off binding for HDAC1/2. These selectivity and kinetic properties differentiate it from HDAC inhibitors utilized in prior rodent behavioral studies, including other benzamides such as the HDAC1/2/3 inhibitor, MS-275, as well as SAHA [3], [19], [28].

### Validation of Cpd-60 as a Histone Deacetylase Inhibitor in Mouse Brain

**Figure 2:**
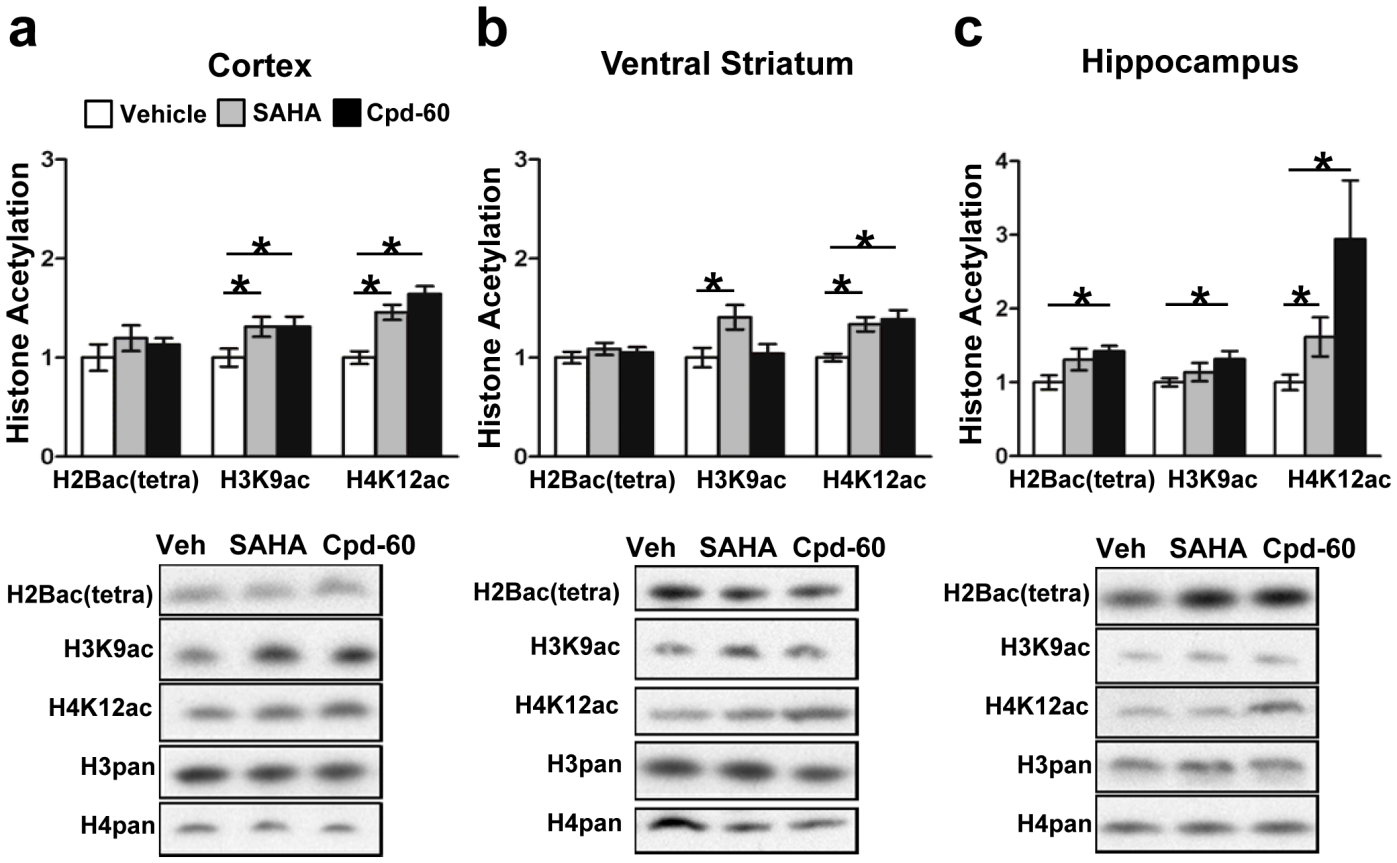
Effects of HDAC inhibitors on histone acetylation in mouse brain. Chronic SAHA (25 mg/kg, i.p.) or Cpd-60 (45 mg/kg, i.p.) significantly increased acetylation of histone H2B(tetra-acetylated), H3K9 and H4K12 in cortex, ventral striatum and hippocampus one hour after the 10^th^ daily treatment (arbitrary units, relative to vehicle control). Representative western blots are shown with total levels of histone H3 (H3pan) and histone H4 (H4pan) used as loading controls. *p<0.05, t-test of Cpd-60 or SAHA versus vehicle. *n = *6 mice/group.

Mood disorder medications, including antidepressants and lithium, typically require prolonged treatment to be effective in patients [30]. We emulated this for *in vivo* experiments using a chronic drug treatment paradigm (>7 days i.p. treatment). To confirm that chronic Cpd-60 and SAHA suppressed HDAC activity in mouse brain, we examined global histone acetylation in cortex, ventral striatum (including NAc) and Hip, regions implicated in regulation of mood-related behaviors [19], [20]. Histone acetylation was measured one hour after chronic treatment, a time point at which both Cpd-60 and SAHA are present in the brain (Fig. 1f). Three histone marks were measured, H2B (‘tetra’acetyl-K5,12,15, and 20), H3K9ac, and H4K12ac, which are associated with active transcription and are sensitive to HDAC2 activity [28], [31]. Consistent with their *in vitro* activity as potent HDAC inhibitors, both Cpd-60 and SAHA significantly increased acetylation in each brain region by 1.5- to 2.0-fold compared to vehicle, indicating both compounds suppress HDAC activity in the brain following systemic administration (Fig. 2). The magnitude of change was consistent with previous studies of mouse brain [3], [6], [14] and suggest that, despite differences in binding kinetics, HDAC subtype selectivity, and pharmacokinetic profiles, chronic treatment with Cpd-60 or SAHA induce similar increases in global histone acetylation in brain regions relevant to mood.

### Chronic Treatment with Cpd-60 Improves Mood-related Behaviors in Mice

**Figure 3:**
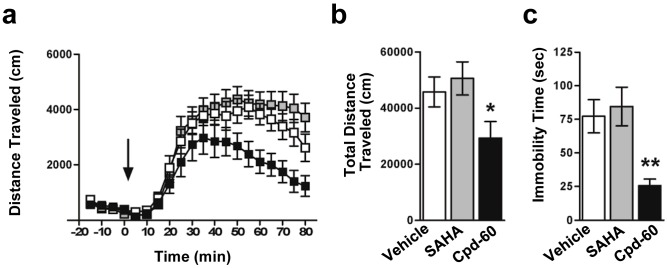
Effect of Cpd-60 treatment on mood-related behaviors in mice. (**a**) Timecourse of locomotor activity in response to and (**b**) total locomotor activity summed over 80 min following acute amphetamine challenge (3.5 mg/kg, i.p.; Time ‘0’ indicated by arrow). Hyperlocomotion in response to amphetamine was significantly reduced in mice chronically treated with Cpd-60 (45 mg/kg, i.p.) but not with SAHA (25 mg/kg, i.p.). (**c**) Time spent immobile in the forced swim test was significantly decreased in mice treated chronically with Cpd-60 but not SAHA compared to vehicle treated control mice. *p<0.05, **p<0.01, ANOVA with Least Significant Difference *post hoc* test.

We next examined the efficacy of Cpd-60 in two established mouse behavioral assays with predictive validity for mood-stabilizer and antidepressant medications; the amphetamine induced hyperlocomotion (AIH) assay and the forced swim test (FST) assay. The AIH assay, in which amphetamine challenge significantly increases locomotor activity, has predictive validity for mood-stabilizing drugs, including lithium and valproate, which significantly attenuate the increased locomotion [16], [26], [32]. Published work from our group has confirmed the attenuating effects of lithium treatment on hyperlocomotion in wild type mice [26]. Following chronic treatment, Cpd-60 (45 mg/kg, i.p.) significantly reduced hyperlocomotion by 36% (F_treatment_ (2,33) = 3.581, *post hoc* p<0.05), whereas SAHA (25 mg/kg, i.p.) had no effect (p = 0.54) compared to vehicle-treated mice (Fig. 3a and b). The attenuating effect of Cpd-60 on hyperlocomotion was not due to non-specific motoric effects, as basal locomotor activity was unchanged preceeding amphetamine challenge (Fig. 3a, t −20 to 0 min) and during test chamber acclimation on the previous day (see Methods; average distance traveled +/− SEM: Cpd-60: 2099 cm +/−249; Vehicle: 1929 cm +/−219). The FST assay is widely-used for evaluating the efficacy of antidepressants, which reduce the time spent immobile in a cylinder of water [33], [34]. Cpd-60 treatment resulted in a significant 67% reduction in immobility time compared to vehicle- or SAHA (Fig. 3C, F_treatment_ (2,33) = 6.588, *post hoc* p<0.01). This indicates an antidepressant-like effect comparable to that previously observed by our group after treatment with lithium (85 mg/kg i.p., 48% reduction, [26]) or the tricyclic antidepressant desipramine (15 mg/kg i.p., 55% reduction, *n = *16/group; data not shown). These results suggest that prolonged inhibition of HDAC1/2 modulates the activity of mood-related neurocircuitry in mice.

### Chronic Cpd-60 Treatment Alters Gene Expression in Brain Circuits Involved in Mood Regulation

Having observed robust behavioral effects following selective HDAC inhibition by Cpd-60, we next sought mechanistic insight into how Cpd-60 modulates molecular pathways regulating mood-related neurocircuitry. As changes in histone acetylation are linked to transcriptional regulation, we examined the effect of chronic HDAC inhibitor treatment on gene expression using whole-genome expression microarrays. PFC, NAc and HIP were profiled one hour after the tenth daily treatment of Cpd-60 or SAHA to investigate transcriptional changes potentially mediated by increased histone acetylation observed at this behaviorally relevant time point (Fig. 2). One-way ANOVA tests revealed that treatment with Cpd-60 or SAHA altered the expression of 4365 transcripts (uncorrected p<0.05). None of the transcripts survived Benjamini-Hochberg false discovery rate (FDR) correction at *q* <0.05 [35], however the microarray results were subsequently verified via quantitative PCR on a subset of genes as detailed below. *Post hoc* testing revealed that, summed across the three brain regions, a similar total number of transcripts were significantly altered by Cpd-60 or SAHA treatment compared to vehicle (Tukey’s HSD p<0.05, 1609 transcripts up- or down-regulated by Cpd-60 versus 1530 by SAHA). These changes reflect a regulatory influence of HDAC inhibitor treatment on only a fraction of the genome (less than 2000 of the >45,000 transcripts assayed by microarray), consistent with previous reports [3], [31].

**Figure 4:**
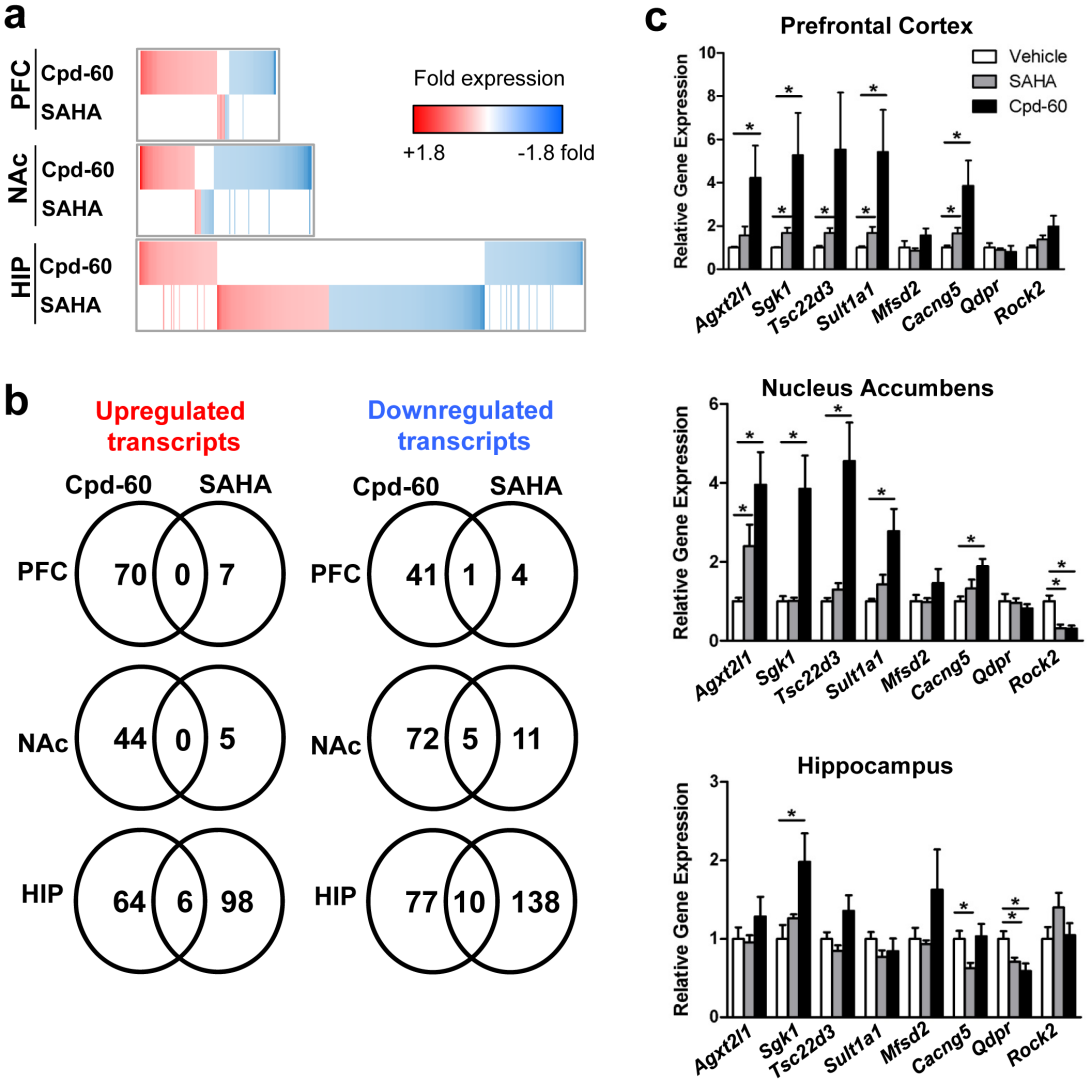
Gene expression changes in mouse brain following chronic HDAC inhibitor treatment. (**a**) Heatmaps illustrating transcript expression changes in mouse brain following chronic HDAC inhibitor treatment for 10 days. Cpd-60 (45 mg/kg, i.p.) significantly upregulated (red) or downregulated (blue) a similar number of transcripts in prefrontal cortex (PFC), nucleus accumbens (NAc), and hippocampus (HIP). Expression changes following SAHA treatment (25 mg/kg, i.p.) were predominantly localized to HIP. (**b**) Venn diagrams illustrate that only 1–10 genes were similarly regulated by Cpd-60 and SAHA treatment depending on brain region. Genes included in heatmaps and Venn diagrams have ≥1.2-fold expression change compared to vehicle (ANOVA p<0.05 with Tukey’s HSD *post hoc* test). (**c**) qPCR validation of a subset of genes with significantly altered expression following Cpd-60 treatment as determined by microarray analysis. *p<0.05, t-test of Cpd-60 or SAHA compared to vehicle.

Focusing on the subset of genes that were altered at least 1.2-fold by Cpd-60 or SAHA compared to vehicle (*post hoc* p<0.05), dramatic expression differences were detected across brain regions and inhibitors. Heatmaps illustrating transcripts upregulated or downregulated by HDAC inhibitor treatment in PFC, NAc or HIP revealed that Cpd-60 and SAHA altered unique sets of genes, indicated by distinct heatmap shading for each inhibitor (Fig. 4a). Large changes in gene expression, shown as intense red/blue shading, were seldom aligned between Cpd-60 and SAHA, illustrating little overlap in transcript regulation. Venn diagrams (Fig. 4b) enumerate transcripts significantly altered by Cpd-60 or SAHA compared to vehicle (≥1.2-fold, *post hoc* p<0.05) and highlight striking differences within each of the three brain regions. Specifically, Cpd-60 treatment altered a similar number of transcripts in each region (44–70 upregulated, 42–87 downregulated), whereas transcripts altered by SAHA were predominantly in the HIP (104 upregulated, 148 downregulated), with far fewer changes detected in the PFC or NAc (7 and 5 upregulated, 5 and 16 downregulated, respectively). Moreover, fewer than ten transcripts were altered by both Cpd-60 and SAHA in any brain region, indicating compound-specific effects on gene expression.

To validate our microarray findings, we applied quantitative real-time PCR (qPCR) to a set of eight genes robustly altered by Cpd-60 versus vehicle (≥1.2-fold, *post hoc* p<0.05). The qPCR results (Fig. 4c) were in agreement with the microarray results for eight of eight genes and provide increased confidence in the microarray gene expression changes. Specifically, the qPCR results supported the microarray results by confirming that Cpd-60 induced robust changes in gene expression, with lesser changes by SAHA, including increases (e.g. *Agxt2l1, Sgk1 and Tsc22d3*) and decreases (e.g. *Qdpr, Rock2*), as well as validation of null effects (e.g. *Mfsd2* in NAc, *Sult1a1* in HIP, Fig.4c; Table S3).

### Cpd-60 Treatment Increases Histone Acetylation at Active Gene Promoter Regions

**Figure 5:**
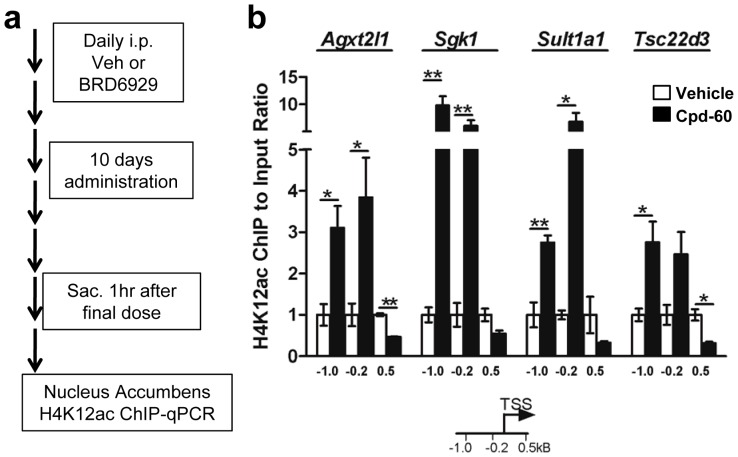
Chromatin immunoprecipitation (ChIP) of nucleus accumbens from Cpd-60 treated mice. (**a**) Schematic of experimental design with 10-day administration of Cpd-60 (45 mg/kg, i.p.). (**b**) Chromatin was immunoprecipitated with an anti-histone H4K12ac-antibody followed by qPCR targeting regions 1.0 or 0.2 kB upstream or 0.5 kB downstream from the transcription start site (TSS). Transcripts upregulated by Cpd-60 treatment had increased histone acetylation at promoter regions upstream, but not downstream, of the TSS in Cpd-60 treated tissue compared to vehicle. *p<0.05, **p<0.01, t-test of Cpd-60 versus vehicle.

We next sought a mechanistic link between the observed increases in histone acetylation and changes in gene expression detected following Cpd-60 treatment. As illustrated by the schematic in Figure 5a, chromatin immunoprecipitation (ChIP) and qPCR were used to examine the histone acetylation status of chromatin surrounding the transcription start site (TSS) of four genes with the greatest microarray expression changes induced by Cpd-60 treatment, alanine-glyoxylate aminotransferase 2-like 1 (*Agxt2l1*), serum/glucocorticoid regulated kinase 1 (*Sgk1*), sulfotransferase family 1A phenol-preferring member 1 (*Sult1a1*), and TSC22 domain family member 3 (*Tsc22d3*). We focused on changes in the NAc as this region integrates dopaminergic and serotonergic neurotransmission – systems that are central to mood regulation [36], [37]. Additionally, a previous study showed that in mice, histone acetylation increases in the NAc correlated with antidepressant-like effects of the HDAC1/2/3 inhibitor MS-275 [3]. We found that H4K12ac was significantly enriched by 2- to 10-fold in Cpd-60-treated mice at regions 0.2 and 1 kb upstream of the TSS of all four genes (Fig. 5b). In contrast, H4K12ac levels were lower than vehicle-treated controls in a region 0.5 kB downstream of each TSS (Fig 5b), indicating that enrichment of H4K12ac at upstream gene promoters was not due to global, non-specific increases in histone acetylation. These data suggest that transcription induced by Cpd-60 results, at least in part, from increased H4K12 acetylation at gene promoter regions.

### Cpd-60 and Lithium Induce Similar Transcriptional Effects in Brain

The behavioral changes observed in the AIH and FST assays following chronic Cpd-60 treatment are similar to those that we and others have found following treatment with lithium [26], [32], as well as valproate in the case of the AIH assay [15]. Lithium and valproate are reported to have synergistic effects on histone acetylation and other molecular targets in mouse models of neurological disease [8], [9], implicating a common mechanism of the two drugs. We therefore reasoned that Cpd-60 and lithium treatments may induce similar effects on the transcriptome. A previous study by McQuillin and colleagues reported that chronic dietary lithium resulted in significant transcriptional changes (up- and down-regulation) of 121 transcripts in mouse brain compared to mice fed a control diet [38]. We therefore used Gene Set Enrichment Analysis (GSEA) to compare the set of genes whose expression was significantly changed by lithium to those we found altered by Cpd-60 or SAHA treatment by microarray analysis (≥1.2-fold change versus vehicle, Tukey’s HSD *post hoc* p<0.05). As McQuillin and colleagues’ study utilized whole brain, we approximated ‘whole brain’ by compiling our microarray expression data from PFC, NAc, and HIP. A subset of 11 of the 390 transcripts altered by Cpd-60 across the three regions were among the 121 modulated by lithium treatment, a statistically significant overlap (FDR-corrected p<0.001), and included the three most upregulated transcripts, *Agxt2l1*, *Sgk1*, and *Tsc22d3* (Table S3). The direction of regulation was the same between Cpd-60 and lithium for 9 of the 11 transcripts. Further, there was no significant overlap between the 288 SAHA-regulated transcripts and those altered by lithium (p = 0.36). We recognize the limitations in comparing these datasets and the small extent of the observed overlap. Nonetheless, these data raise the intriguing possibility that common transcriptional regulatory mechanisms between Cpd-60 and lithium may underlie their similar effects on mood-related behaviors in mice.

## Discussion

Research in post-mortem human brain and preclinical models has provided evidence that chromatin-mediated neuroplasticity likely plays an important role in the mechanisms underlying psychiatric diseases and clinical treatments. A major finding of this study is that chronic systemic treatment of mice with Cpd-60, a slow-binding, selective inhibitor of HDAC1 and HDAC2, modulates brain function as demonstrated by behavioral alterations in paradigms with predictive validity for mood-stabilizing drugs and antidepressants. These alterations were associated with significant transcriptional changes in mouse PFC, NAc and HIP, brain regions involved in regulating mood, that were mediated by epigenetic modifications as evidenced by increased histone acetylation at the promoter regions of upregulated transcripts. Comparatively, the class I and II HDAC inhibitor, SAHA, did not influence mouse behavior in either paradigm and transcriptional changes were predominantly localized to the hippocampus despite evidence of HDAC inhibition in all brain regions examined. These findings underscore the need to understand the mechanisms by which HDAC subtypes regulate histone acetylation and gene transcription, which in turn modulate brain function, as well as how highly-selective HDAC inhibitors can modulate chromatin-mediated neuroplasticity and inform treatment design.

Aside from different HDAC selectivity profiles of Cpd-60 and SAHA, other potential explanations for the distinct behavioral and molecular effects we observed may be related to brain exposure and binding kinetics. Systemic Cpd-60 treatment resulted in peak brain concentrations within 5 min and remained >0.1 µM for at least 8 hr. In contrast, systemic SAHA treatment, although reaching peak concentrations quickly, was cleared from the brain within 2 hr. Nevertheless, SAHA and other HDAC inhibitors are reported to alter mood-related behaviors when infused directly into the brain [3], [19], suggesting that neuroplasticity and behavior can be altered if brain levels of HDAC inhibitors are sufficiently high. The slow binding kinetics of Cpd-60 also support that sustained inhibition of HDAC enzymes may be potentially beneficial in neuropsychiatric disease treatment models.

A limitation to interpreting the behavioral and molecular effects of HDAC inhibitor treatment is that, while the expression of HDAC subtypes has been examined using harvested brain [39], [40], identifying the neuroanatomical distribution and function of HDAC subtypes in living animals remains a lasting challenge. Emerging *in vivo* imaging research investigating radiolabeled HDAC inhibitor compounds [29] may provide tools to reveal the distribution of HDAC subtypes and, ideally, enzymatic activity in the human brain. Meanwhile, this work helps define the relationship between blood-brain barrier (BBB) penetration of compounds and their efficacy in the treatment of CNS disorders. As such, rodent and non-human primate brain imaging data indicate that systemically delivered MS-275 is limited by poor BBB permeability [29], despite altering mood-related behaviors in mice following direct brain infusion [3], [19]. Future studies aimed at identifying HDAC inhibitors with improved selectivity, pharmacokinetics, and BBB permeability, as well as those clarifying the functional distribution of HDAC subtypes in brain, will be critical to develop HDAC therapies for the CNS.

Insight into the mechanism underlying behavioral changes induced by chronic Cpd-60 treatment can be gained from transcriptional changes observed in brain. We interpret the robust gene expression changes detected in this study as suggesting a role of promoter region histone acetylation in the selective transcriptional activation of genes that functionally regulate mood-related neurocircuitry. In our working model, chronic suppression of HDAC1/2 activity by Cpd-60 treatment alters promoter-region histone acetylation of a subset of genes, opening chromatin structure that facilitates transcriptional response to homeostatic cues and regulatory proteins. Resulting gene expression changes converge and, based on our behavioral evidence, function to insulate against challenges to mood-related neurocircuitry.

Based on our microarray data, such homeostatic cues may include glucocorticoid signaling, a stress-response system altered in mood disorders [41], [42] and previously implicated in chromatin-mediated neuroplasticity changes underlying mood-related behaviors [43]. Lead examples for this model based on the most significant Cpd-60-induced expression changes include *Sgk1* and *Sult1a1*, both induced by glucocorticoid signaling [44], [45]. *Sgk1* and *Sult1a1* were also among the genes reported to be upregulated in rodent brain following lithium treatment [38], and also by diverse antidepressant therapies [46]. Further, *Sgk1* was increased in rat brain after administration of the antipsychotic clozapine [47]. In addition, its protein product SGK phosphorylates and deactivates glycogen synthase kinase-3 (GSK-3), a known target of lithium [48], suggesting that protein effectors in the GSK-3/Wnt signaling pathway (e.g. β-catenin) may mediate transcriptional changes initiated by treatment with Cpd-60, lithium or other drugs that alleviate mood dysregulation. Finally, clozapine and lithium require SGK to suppress nuclear localization of the eukaryotic transcription factor FOXO [49]. Together, these data support that the transcriptional effects we observed in Cpd-60-treated mice may result in part from a glucocorticoid signaling cascade that incorporates SGK1 and modulates the activity of transcription factor proteins such as β-catenin and FOXO.

Importantly, we point out that understanding the impact of selective HDAC inhibitor treatment on behavioral response would benefit from examination in additional tests and paradigms. However, we found that treatment with Cpd-60 (45 mg/kg, i.p.) for longer than 10 days was not well tolerated resulting in compromised health that precluded more thorough behavioral characterization. We did not observe any health compromise in mice treated with SAHA (25 mg/kg, i.p.) for the same time period. Indeed, this is a major caveat in considering compounds like Cpd-60 in designing new therapies for mental health disorders where long-term treatment is often required for efficacy. However, using these and related HDAC inhibitor tool compounds in basic research, future studies hold promise to resolve how HDAC subtype affinity, brain penetrance and clearance relate to both beneficial and deleterious effects of HDAC inhibition. To this end, it is interesting to consider the impact of selective HDAC1/2 inhibition on CNS disease models including chronic corticosteroid exposure [50], as well as chemical and genetic models of neurodegenerative disease [51]–[53]. Likewise, emerging compounds such as a highly selective HDAC3 inhibitor recently used in a study of addiction-related memory [54] will be important in further describing the role of HDAC enzymes in mood-related behaviors and how these enzymes may be best targeted in clinical drug development.

We recognize that, although poorly understood, acetylation of non-histone proteins likely plays a part in HDAC inhibitor effects [55]. While Cpd-60 does not functionally inhibit either HDAC5 or HDAC6, reports have indicated a role for the non-histone targets of these class II HDACs in regulating emotional behavior, stress and GC signaling [56]–[58]. Comparing the behavioral and molecular responses in these reports to those induced by selective class I HDAC inhibitors will clarify the role of acetylation of histone and non-histone proteins to coordinate changes in chromatin structure and mood-related neurocircuitry. Toward this aim, the aggregate changes induced by Cpd-60 treatment represent a signature of gene expression that could be used to discriminate critical factors induced by clinical drugs or other chromatin modifying compounds that may influence psychiatric disease symptoms and treatment. Studies to address this and other mechanistic questions are ongoing.

In conclusion, efforts in genetic and epigenetic research will continue to discover and refine the mechanistic underpinnings of these diseases and lead to targeted, mechanism-based treatments. Our current findings stand as supportive evidence that selective inhibition of HDAC1 and HDAC2 results in beneficial changes in neuroplasticity and may be a novel target for mood disorder therapy. Overall, the results – and limitations – of this study underscore the importance of developing brain-penetrant selective HDAC inhibitors as well as the challenges related of long term, systemic treatment with HDAC inhibitors. Continued research on small molecule, selective HDAC inhibitors will advance understanding of improvements that can made in engineering chromatin modifying drugs and how these may be best applied in treating clinical brain dysfunction.

## Supporting Information

Table S1 **CNS Target Binding Assay.**
(PDF)

Table S2 **Primer sequences used for qPCR and ChIP experiments.**
(PDF)

Table S3 **Significant overlap in gene expression changes associated with chronic Cpd-60 or lithium treatment.** Transcript microarray data expressed as fold change relative to vehicle controls. Eleven of 368 transcripts altered by Cpd-60 in PFC, NAc or HIP (>1.2-fold vs. vehicle, bold text indicates post hoc p<0.05 by Tukey’s HSD) were among 121 transcripts modulated in whole brain of mice treated chronically with lithium (right panel, **McQuillin et al, 2007); overlap significant at p<0.001 by Gene Set Analysis. Gray shading highlights genes upregulated by Cpd-60 and lithium, with lesser effects by SAHA.
(PDF)

Methods S1 **Supplemental Materials and Methods.**
(DOCX)
