# Supplementary material for: A Selective HDAC 1/2 Inhibitor Modulates Chromatin and Gene Expression in Brain and Alters Mouse Behavior in Two Mood-Related Tests
Source: PLoS One. 2013 Aug 14;8(8):e71323. doi: 10.1371/journal.pone.0071323 (PMC3743770; doi:10.1371/journal.pone.0071323)
Supplement: Table S1 — CNS Target Binding Assay. (PDF) [file pone.0071323.s001.pdf]

Table S1: CNS Target Binding Assay

Receptor Assay	Origin	Ligand	Concentration	Non Specific	Incubation
Adenosine $_1$ (antagonist radioligand)	human recombinant (CHO cells)	$[^3H]$DPCPX	1 nM	DPCPX (1 µM)	60 min./22°C
Adenosine $_{2A}$ (agonist radioligand)	human recombinant (HEK-293 cells)	$[^3H]$CGS 21680	6 nM	NECA (10 µM)	120 min./22°C
Adenosine $_3$ (agonist radioligand)	human recombinant (HEK-293 cells)	$[^{125}I]$AB-MECA	0.15 nM	IB-MECA (1 µM)	120 min./22°C
Adrenergic α_1 (non-selective) (antagonist radioligand)	rat cerebral cortex	$[^3H]$prazosin	0.25 nM	prazosin (0.5 µM)	60 min./22°C
Adrenergic α_2 (non-selective) (antagonist radioligand)	rat cerebral cortex	$[^3H]$RX 821002	0.5 nM	(-)epinephrine (100 µM)	60 min./22°C
Adrenergic β_1 (agonist radioligand)	human recombinant (HEK-293 cells)	$[^3H]$(-)CGP 12177	0.15 nM	alprenolol (50 µM)	60 min./22°C
Adrenergic β_2 (agonist radioligand)	human recombinant (CHO cells)	$[^3H]$(-)CGP 12177	0.2 nM	alprenolol (50 µM)	120 min./22°C
Angiotensine -$_1$ (antagonist radioligand)	human recombinant (HEK-293 cells)	$[^{125}I][Sar^1,Ile^8]$-AT-II	0.05 nM	angiotensin-II (10 µM)	120 min./37°C
Angiotensine-$_2$ (antagonist radioligand)	human recombinant (CHO cells)	$[^{125}I]$CGP 42112A	0.04 nM	angiotensin-II (1 µM)	180 min./37°C
Benzodiazepine (central) (agonist radioligand)	rat cerebral cortex	$[^3H]$flunitrazepam	0.4 nM	diazepam (3 µM)	60 min./4°C
Benzodiazepine (peripheral) (antagonist radioligand)	rat heart	$[^3H]$PK 11195	0.2 nM	PK 11195 (10 µM)	15 min./22°C
Bombesin (non-selective) (agonist radioligand)	rat cerebral cortex	$[^{125}I][Tyr^4]$bombesin	0.01 nM	bombesin (1 µM)	60 min./22°C
Bradykinin (agonist radioligand)	human recombinant (CHO cells)	$[^3H]$bradykinin	0.2 nM	bradykinin (1 µM)	60 min./22°C
Calcitorin gene-related peptide (agonist radioligand)	human recombinant (CHO cells)	$[^{125}I]$hCGRPα	0.03 nM	hCGRPα (1 µM)	90 min./22°C
Cannabinoid (agonist radioligand)	human recombinant (CHO cells)	$[^3H]$CP 55940	0.5 nM	WIN 55212-2 (10 µM)	120 min./37°C
Cholecystokinin-A (agonist radioligand)	human recombinant (CHO cells)	$[^{125}I]$CCK-8s	0.08 nM	CCK-8s (1 µM)	60 min./22°C
Cholecystokinin-B (agonist radioligand)	human recombinant (CHO cells)	$[^{125}I]$CCK-8s	0.08 nM	CCK-8s (1 µM)	60 min./22°C
Dopamine-D_1 (antagonist radioligand)	human recombinant (CHO cells)	$[^3H]$SCH 23390	0.3 nM	SCH 23390 (1 µM)	60 min./22°C
Dopamine-D_{2S} (antagonist radioligand)	human recombinant (HEK-293 cells)	$[^3H]$spiperone	0.3 nM	(+)butaclamol (10 µM)	60 min./22°C
Dopamine-D_3 (antagonist radioligand)	human recombinant (CHO cells)	$[^3H]$spiperone	0.3 nM	(+)butaclamol (10 µM)	60 min./22°C

Receptor Assay	Origin	Ligand	Concentration	Non Specific	Incubation
Dopamine-$D_{4.4}$ (antagonist radioligand)	human recombinant (CHO cells)	$[^3H]$spiperone	0.3 nM	(+)butaclamol (10 µM)	60 min./22°C
Dopamine-D_5 (antagonist radioligand)	human recombinant (GH4 cells)	$[^3H]$SCH 23390	0.3 nM	SCH 23390 (10 µM)	60 min./22°C
Endothelin -ET_A (agonist radioligand)	human recombinant (CHO cells)	$[^{125}I]$endothelin-1	0.03 nM	endothelin-1 (0.1 µM)	120 min./37°C
Endothelin -ET_B (agonist radioligand)	human recombinant (CHO cells)	$[^{125}I]$endothelin-1	0.03 nM	endothelin-1 (0.1 µM)	120 min./37°C
GABA (non-selective) (agonist radioligand)	rat cerebral cortex	$[^3H]$GABA	10 nM	GABA (100 µM)	60 min./22°C
$Galanin_1$ (agonist radioligand)	human recombinant (HEK-293 cells)	$[^{125}I]$galanin	0.1 nM	galanin (1 µM)	60 min./22°C
$Galanin_2$ (agonist radioligand)	human recombinant (CHO cells)	$[^{125}I]$galanin	0.05 nM	galanin (1 µM)	120 min./22°C
Growth Factors - PDGF (agonist radioligand)	Balb/c 3T3 cells	$[^{125}I]$PDGF BB	0.03 nM	PDGF BB (10 nM)	180 min./4°C
Chemokines (IL-8B) (agonist radioligand)	human recombinant (HEK-293 cells)	$[^{125}I]$IL-8	0.025 nM	IL-8 (30 nM)	60 min./22°C
TNF-α (agonist radioligand)	U-937 cells	$[^{125}I]$TNF-α	0.1 nM	TNF-α (10 nM)	120 min./4°C
Chemokines - CCR1 (agonist radioligand)	human recombinant (HEK-293 cells)	$[^{125}I]$MIP-1α	0.02 nM	MIP-1α (100 nM)	120 min./22°C
Histamine-H_1 (antagonist radioligand)	human recombinant (CHO cells)	$[^3H]$pyrilamine	3 nM	pyrilamine (1 µM)	60 min./22°C
Histamine-H_2 (antagonist radioligand)	human recombinant (CHO cells)	$[^{125}I]$APT	0.075 nM	tiotidine (100 µM)	120 min./22°C
Melanocortin - MC_4 (agonist radioligand)	human recombinant (CHO cells)	$[^{125}I]$NDP-α-MSH	0.05 nM	NDP-α-MSH (1 µM)	120 min./37°C
Melatorin (ML_{1A}) (agonist radioligand)	human recombinant (CHO cells)	$[^{125}I]$2-iodomelatonin	0.025 nM	melatonin (1 µM)	60 min./22°C
Muscarinic-M_1 (antagonist radioligand)	human recombinant (CHO cells)	$[^3H]$pirenzepine	2 nM	atropine (1 µM)	60 min./22°C
Muscarinic-M_2 (antagonist radioligand)	human recombinant (CHO cells)	$[^3H]$AF-DX 384	2 nM	atropine (1 µM)	60 min./22°C
Muscarinic-M_3 (antagonist radioligand)	human recombinant (CHO cells)	$[^3H]$4-DAMP	0.2 nM	atropine (1 µM)	60 min./22°C
Muscarinic-M_4 (antagonist radioligand)	human recombinant (CHO cells)	$[^3H]$4-DAMP	0.2 nM	atropine (1 µM)	60 min./22°C
Muscarinic-M_5 (antagonist radioligand)	human recombinant (CHO cells)	$[^3H]$4-DAMP	0.3 nM	atropine (1 µM)	60 min./22°C
Neurokinin-NK_1 (agonist radioligand)	U-373MG cells (endogenous)	$[^{125}I]$BH-SP	0.15 nM	$[Sar^9,Met(O_2)^{11}]$-SP (1 µM)	60 min./22°C
Neurokinin-NK_2 (agonist radioligand)	human recombinant (CHO cells)	$[^{125}I]$NKA	0.1 nM	$[Nleu^{10}]$-NKA (4-10) (10 µM)	60 min./22°C

Receptor Assay	Origin	Ligand	Concentration	Non Specific	Incubation
Neurokinin-NK_3 (antagonist radioligand)	human recombinant (CHO cells)	$[^3H]$SR 142801	0.4 nM	SB 222200 (10 µM)	120 min./22°C
Neuropeptide Y-Y_1 (agonist radioligand)	SK-N-MC cells (endogenous)	$[^{125}I]$peptide YY	0.025 nM	NPY (1 µM)	120 min./37°C
Neuropeptide Y-Y_2 (agonist radioligand)	KAN-TS cells	$[^{125}I]$peptide YY	0.015 nM	NPY (1 µM)	60 min./37°C
Neurotensin (NT_1) (agonist radioligand)	human recombinant (CHO cells)	$[^{125}I]Tyr^3$-neurotensin	0.05 nM	neurotensin (1 µM)	60 min./4°C
δ_2 Opioid (DOP) (agonist radioligand)	human recombinant (CHO cells)	$[^3H]$DADLE	0.5 nM	naltrexone (10 µM)	120 min./22°C
κ Opioid (KOP) (agonist radioligand)	rat recombinant (CHO cells)	$[^3H]$U 69593	1 nM	naloxone (10 µM)	60 min./22°C
µ Opioid (MOP) (agonist radioligand)	human recombinant (HEK-293 cells)	$[^3H]$DAMGO	0.5 nM	naloxone (10 µM)	120 min./22°C
Opioid-like (ORL1) (agonist radioligand)	human recombinant (HEK-293 cells)	$[^3H]$nociceptin	0.2 nM	nociceptin (1 µM)	60 min./22°C
Vasoactive intestinal peptide PAC_1 (PACAP) (agonist radioligand)	human recombinant (CHO cells)	$[^{125}I]PACAP_{1-27}$	0.015 nM	$PACAP_{1-27}$ (100 nM)	120 min./22°C
Non-steroid nuclear receptor PPARγ (agonist radioligand)	human recombinant (*E. coli*)	$[^3H]$rosiglitazone	5 nM	rosiglitazone (10 µM)	120 min./4°C
Glutamate - PCP (antagonist radioligand)	rat cerebral cortex	$[^3H]$TCP	10 nM	MK 801 (10 µM)	120 min./37°C
Prostanoid - EP_4 (agonist radioligand)	human recombinant (CHO cells)	$[^3H]PGE_2$	1 nM	PGE_2 (10 µM)	120 min/22°C
Prostanoid - TP (TXA_2/PGH_2) (antagonist radioligand)	human recombinant (HEK-293 cells)	$[^3H]$SQ 29548	5 nM	U 44069 (10 µM)	60 min./22°C
Prostanoid - IP (PGI_2) (agonist radioligand)	human recombinant (HEK-293 cells)	$[^3H]$iloprost	10 nM	iloprost (10 µM)	60 min./22°C
Purinergic-P2X (agonist radioligand)	rat urinary bladder	$[^3H]$α,β-MeATP	3 nM	α,β-MeATP (10 µM)	120 min./4°C
Purinergic-P2Y (agonist radioligand)	rat cerebral cortex	$[^{35}S]$dATPαS	10 nM	dATPαS (10 µM)	60 min./22°C
Serotonin 5-HT_{1A} (agonist radioligand)	human recombinant (HEK-293 cells)	$[^3H]$8-OH-DPAT	0.3 nM	8-OH-DPAT (10 µM)	60 min./22°C
Serotonin 5-HT_{1B} (antagonist radioligand)	rat cerebral cortex	$[^{125}I]$CYP (+ 30 µM (-)propranolol)	0.1 nM	serotonin (10 µM)	120 min./37°C
Serotonin 5-HT_{2A} (antagonist radioligand)	human recombinant (HEK-293 cells)	$[^3H]$ketanserin	0.5 nM	ketanserin (1 µM)	60 min./22°C
Serotonin 5-HT_{2B} (agonist radioligand)	human recombinant (CHO cells)	$[^{125}I]$(±)DOI	0.2 nM	(±)DOI (1 µM)	60 min./22°C
Serotonin 5-HT_{2C} (antagonist radioligand)	human recombinant (CHO cells)	$[^3H]$mesulergine	1 nM	RS 102221 (10 µM)	60 min./37°C
Serotonin 5-HT_3 (antagonist radioligand)	human recombinant (CHO cells)	$[^3H]$BRL 43694	0.5 nM	MDL 72222 (10 µM)	120 min./22°C

Receptor Assay	Origin	Ligand	Concentration	Non Specific	Incubation
Serotonin 5-HT_{5A} (agonist radioligand)	human recombinant (CHO cells)	$[^3H]$LSD	1 nM	serotonin (100 µM)	60 min./37°C
Serotonin 5-HT_6 (agonist radioligand)	human recombinant (CHO cells)	$[^3H]$LSD	2 nM	serotonin (100 µM)	120 min./37°C
Serotonin 5-HT_7 (agonist radioligand)	human recombinant (CHO cells)	$[^3H]$LSD	4 nM	serotonin (10 µM)	120 min./22°C
σ Sigma (non-selective) (agonist radioligand)	rat cerebral cortex	$[^3H]$DTG	8 nM	haloperidol (10 µM)	120 min./22°C
Somatostatin (non-selective) (agonist radioligand)	AtT-20 cells	$[^{125}I]Tyr^{11}$-somatostatin-14	0.05 nM	somatostatin-14 (300 nM)	60 min./37°C
Steroid nuclear receptor GR (agonist radioligand)	IM-9 cells (cytosol)	$[^3H]$dexamethasone	1.5 nM	triamcinolone (10 µM)	6 h./4°C
Vasoactive intestinal peptide (VIP_1) (agonist radioligand)	human recombinant (CHO cells)	$[^{125}I]$VIP	0.04 nM	VIP (1 µM)	60 min./22°C
Vasopressin - V_{1a} (agonist radioligand)	human recombinant (CHO cells)	$[^3H]$AVP	0.3 nM	AVP (1 µM)	60 min./22°C
Ca^{2+} channel (L, verapamil site) (phenylalkylamine) (antagonist radioligand)	rat cerebral cortex	$[^3H]$(-)D 888	3 nM	D 600 (10 µM)	120 min./22°C
Potassium- K_V channel (antagonist radioligand)	rat cerebral cortex	$[^{125}I]$α–dendrotoxin	0.01 nM	α–dendrotoxin (50 nM)	60 min./22°C
Potassium- SK_{Ca} channel (antagonist radioligand)	rat cerebral cortex	$[^{125}I]$apamin	0.007 nM	apamin (100 nM)	60 min./4°C
Na^+ channel (site 2) (antagonist radioligand)	rat cerebral cortex	$[^3H]$batrachotoxinin	10 nM	veratridine (300 µM)	60 min./22°C
Cl^- channel (GABA-gated) (antagonist radioligand)	rat cerebral cortex	$[^{35}S]$TBPS	3 nM	picrotoxinin (20 µM)	120 min./22°C
norepinephrine transporter (antagonist radioligand)	human recombinant (CHO cells)	$[^3H]$nisoxetine	1 nM	desipramine (1 µM)	120 min./4°C
dopamine transporter (antagonist radioligand)	human recombinant (CHO cells)	$[^3H]$BTCP	4 nM	BTCP (10 µM)	120 min./4°C
Serotonin transporter (antagonist radioligand)	human recombinant (CHO cells)	$[^3H]$imipramine	2 nM	imipramine (10 µM)	60 min./22°C
