# Supplementary material for: A Selective HDAC 1/2 Inhibitor Modulates Chromatin and Gene Expression in Brain and Alters Mouse Behavior in Two Mood-Related Tests
Source: PLoS One. 2013 Aug 14;8(8):e71323. doi: 10.1371/journal.pone.0071323 (PMC3743770; doi:10.1371/journal.pone.0071323)
Supplement: Table S2 — Primer sequences used for qPCR and ChIP experiments. (PDF) [file pone.0071323.s002.pdf]

Table S2: Primer sequences used for qPCR and ChIP experiments

Gene name	Forward (5'-3')	Reverse (5'-3')
Agxt2l1	GCCCAGCATTCAGGGTTGGGG	AGGTGTTCGGAGGAGGGGCC
Sgk1	GCAGCCGTATGACCGGACGG	AAACGGGGGCAGGCCGTAGA
Tsc22d3	AGCAGGCCATGGACCTCGTGA	CGCGCTCCAGCTGGGAGTTC
Sult1a1	TGGGAAAGTGTCCTATGGGTCGTGG	ACAGGGTGAGTGCGTCTCAGCT
Mfsd2	TGCCCTTGCACTGAGCAGCTG	GCAGCCCTAAGGCTTGGCCC
Cacng5	CCCTCCACAGCCACAGCCAC	CGCCGCCCTCACATCACAGG
Qdpr	ATCCTTTTGTGTTCCAGCCTAGGC	AGGAAGAACACTCCAAGCACTCACA
Rock2	TCCCAACCAACTGTGAGGCATGT	TGTGGCACCTACGGCACTCT
**Primers for ChIP**	**1.0kB upstream (forward)**	**1.0kB upstream (reverse)**
Agxt2l1	AGAACCCAAAGCTCCCGGCG	GCCTGACTCCCCTCAGGCTGG
Sgk1	ACGGACGGGGTTTAAGGCAGTG	CGACCTGGAGTGACCCTTGCG
Sult1a1	TGGCATCTTGGCTCCTCTCGC	GAGTGGGGTTGGGGGATGCG
Tsc22d3	GCCAAGGCTGGGGCAAGGAG	GGCCCCTAAGGTCCCAGGCA
**Primers for ChIP**	**0.2kB upstream (forward)**	**0.2kB upstream (reverse)**
Agxt2l1	TGGGGAGGAGGAAGCAAGGCA	TCTGCAGATGTCCCCTGCCCC
Sgk1	CGGGACACCGAGGAGGGGAG	GGAGGAGGGCACTGGAGGGG
Sult1a1	TGACGTCAGAGGTTCCAGGCA	AGTAGCCAGATGGGGACACCTGC
Tsc22d3	TGGCATTTCCTCCTCTCGACG	AGCTCTGTCGTCCCTCCCCA
**Primers for ChIP**	**0.5kB downstream (forward)**	**0.5kB downstream (reverse)**
Agxt2l1	CATCATGTGATTGATGCCTCCCCA	AGCCTCACTTTTGCCCGGGG
Sgk1	GCCAGCAACACCTATGCATGCA	CCCGCCCCATGAAGTCTTCGC
Sult1a1	TCAGTTCTAGATGGGCTGAGGCATT	AGTGCAGCTTCAGGCCATGGC
Tsc22d3	TGCCTGGGACCTTAGGGGCC	TGAGCGGCCTGCTTTTGGGC
