# Supplementary material for: A Selective HDAC 1/2 Inhibitor Modulates Chromatin and Gene Expression in Brain and Alters Mouse Behavior in Two Mood-Related Tests
Source: PLoS One. 2013 Aug 14;8(8):e71323. doi: 10.1371/journal.pone.0071323 (PMC3743770; doi:10.1371/journal.pone.0071323)
Supplement: Table S3 — Significant overlap in gene expression changes associated with chronic Cpd-60 or lithium treatment. Transcript microarray data expressed as fold change relative to vehicle controls. Eleven of 368 transcripts altered by Cpd-60 in PFC, NAc or HIP (>1.2-fold vs. vehicle, bold text indicates post hoc p<0.05 by Tukey’s HSD) were among 121 transcripts modulated in whole brain of mice treated chronically with lithium (right panel, **McQuillin et al, 2007); overlap significant at p<0.001 by Gene Set Analysis. Gray shading highlights genes upregulated by Cpd-60 and lithium, with lesser effects by SAHA. (PDF) [file pone.0071323.s003.pdf]

Table S3: Significant overlap in gene expression changes associated with chronic Cpd-60 or lithium treatment.

Transcript microarray data expressed as fold change relative to vehicle controls. Eleven of 368 transcripts altered by Cpd-60 in PFC, NAc or HIP (>1.2-fold vs. vehicle, bold text indicates post hoc $p<0.05$ by Tukey's HSD) were among 121 transcripts modulated in whole brain of mice treated chronically with lithium (right panel, **McQuillin et al, 2007); overlap significant at $p<0.001$ by Gene Set Analysis. Gray shading highlights genes upregulated by Cpd-60 and lithium, with lesser effects by SAHA.

Gene Symbol	Region	Cpd-60	SAHA	Lithium**
Agxt2l1	PFC	-	-	
	NAc	**1.77**	1.44	2.73
	HIP	1.45	1.04	
Sgk1	PFC	1.51	1.05	
	NAc	1.68	1.08	2.85
	HIP	**1.85**	1.01	
Tsc22d3	PFC	**1.54**	1.02	
	NAc	**1.80**	1.10	1.95
	HIP	**1.51**	1.25	
Sult1a1	PFC	**1.28**	1.01	
	NAc	1.33	1.09	6.28
	HIP	1.12	1.00	
Mfsd2	PFC	**1.39**	1.02	
	NAc	-	-	2.65
	HIP	**1.46**	1.20	
Usp54	PFC	**1.22**	1.05	
	NAc	-	-	1.57
	HIP	-	-	
Nrxn1	PFC	**1.20**	1.19	
	NAc	-	-	-1.18
	HIP	-	-	
Qdpr	PFC	-	-	
	NAc	-	-	-1.46
	HIP	**-1.22**	**-1.27**	
Igfbp5	PFC	-	-	
	NAc	-	-	-1.56
	HIP	**-1.27**	-1.11	
Mef2c	PFC	-	-	
	NAc	**-1.20**	-1.12	-1.22
	HIP	-	-	
Per2	PFC	**-1.31**	-1.11	
	NAc	-	-	2.47
	HIP	-	-	
