# Supplementary material for: A Selective HDAC 1/2 Inhibitor Modulates Chromatin and Gene Expression in Brain and Alters Mouse Behavior in Two Mood-Related Tests
Source: PLoS One. 2013 Aug 14;8(8):e71323. doi: 10.1371/journal.pone.0071323 (PMC3743770; doi:10.1371/journal.pone.0071323)
Supplement: Methods S1 — Supplemental Materials and Methods. (DOCX) [file pone.0071323.s004.docx]

**Supplemental Materials and Methods**

Animals

C57BL/6 male mice were purchased at 10 weeks of age from The Jackson Laboratory and Taconic Farms. Mice were housed on a diurnal 12:12-h light-dark cycle, four per cage, with free access to food and water. All animals were acclimated to the colony for one week before procedures. All mouse procedures were approved by the Massachusetts Institute of Technology Animal Care and Use Committee. A female (*Papio anubis)* baboon was used to determine the brain uptake and pharmacokinetics of carbon-11 labeled Cpd-60. This study was conducted at Brookhaven National Laboratory where it was approved by the Brookhaven Institutional Animal Care and Use Committee. All animal studies followed the National Institutes of Health Guide for the Care and Use of Laboratory Animals.

Pharmacokinetic Profile Determination

Plasma and brain samples and dose formulations were analyzed as previously described [[1](#_ENREF_1)] using high performance liquid chromatography/mass spectrometry consisting of an Agilent liquid chromatograph equipped with an 1100 series isocratic pump, autosampler and degasser (Agilent Technologies Inc.,USA), and an ABI Inc. API3000 triple-quadpole mass spectrometer with ESI interface (ABI Inc, Canada). Data acquisition and control system were created using Analyst 1.4 software from ABInc.

CNS Target Binding Assays

Binding of Cpd-60 to 80 common CNS targets was analysed using the High-Thoughput Profile P-3 panel (Cerep, France). The specific ligand binding to the receptors is defined as the difference between the total binding and the nonspecific binding determined in the presence of an excess of unlabelled ligand. In each experiment, the respective reference compound was tested concurrently with Cpd-60 (10µM) in duplicate and the data were compared with historical values determined at Cerep. The results were expressed as a percent of control specific binding ((measured specific binding/control specific binding) x 100) and as a percent inhibition of control specific binding (100-((measured specific binding/control specific binding) x 100)) obtained in the presence of Cpd-60. The experiment was accepted in accordance with Cerep’s validation Standard Operating Procedure.

Biochemical Assays

HDAC activity was measured *in vitro* using recombinant human HDACs 1-9 using the Caliper EZ reader II system (Caliper Life Sciences, USA) in a microfluidic lab-on-chip technology assay with reaction rates normalized to the average of DMSO control treatments. All HDACs were purchased from BPS Bioscience, substrates were synthesized in house, and all the other reagents purchased from Sigma.

For HDAC inhibition assays, purified HDACs were incubated with 2 μM carboxyfluorescein
(FAM)-labeled acetylated peptide substrate and test compound for 60 min at room temperature in HDAC assay buffer containing 50 mM HEPES (pH 7.4), 100 mM KCl, 0.01% BSA and 0.001% Tween-20. Reactions (in duplicate) were terminated by addition of the known pan-HDAC inhibitor LBH-589 (panobinostat) with a final concentration of 1.5 μM. Substrate and product were separated electrophoretically and fluorescence intensity in the substrate and product peaks determined and analyzed by Caliper EZ reader II. The percent inhibition was plotted against the compound concentration and the IC50 value determined from the logistic dose-response curve fitting by Origin 8.0 software [[2](#_ENREF_2)]

Binding kinetics of Cpd-60 and SAHA with HDACs 1, 2, and 3 were evaluated by progression curves in inhibition and dilution experiments as previously described [[3](#_ENREF_3)]. Kinetic reactions were assembled in microtiter plate wells by adding 1.7 nM, 1.7 nM and 0.5 nM of HDACs 1, 2, and 3, respectively, into separate reaction mixtures containing 2 μM of a fluorescently labeled, acetylated histone H4K12 (AcH4K12) tripeptide substrate that was synthesized in-house. Plates were immediately placed into a Caliper LabChip EZ Reader and the wells sampled periodically throughout a 3hr reaction period. The fluorescent product and substrate were separated and monitored on the Caliper microfluidic instrument and the conversion of substrate calculated with Caliper software. Progression curves at different HDAC inhibitor concentrations were analyzed with a nonlinear regression program in Origin 8.5 to the integrated rate equation for fast- or slow-binding inhibitors. Off rates were determined from a dilution experiment in which HDAC1 (0.1 μM), HDAC2 (0.1 μM) or HDAC3 (0.05 μM) were incubated with Cpd-60 or SAHA at 0.1 μM, 0.5 μM or 25 μM respectively for 1 hour. The mixtures were diluted 100-fold into buffer containing 2 μM of the AcH4K12 substrate. HDAC-diluted samples were measured continuously over 4hr. HDAC inhibitor off-rates were calculated by measuring HDAC activity of the inhibitor-preincubated HDAC compared to a non-preincubated control.

Mouse Primary Neuronal Histone Acetylation Assays

Mouse primary neuronal cultures were generated in factory precoated poly-D-lysine 96-well plates (BD Biosciences #BD356692) treated overnight with 75 μL/well of laminin [0.05 mg/mL] (Sigma #L2020) in PBS buffer. E15 embryonic mouse forebrain was dissociated into a single cell suspension by gentle trituration following trypsin/DNAse digestion (trypsin: Cellgro #25-052-Cl; DNAse: Sigma #D4527). 12,500 cells per well were plated in 100 μL Neurobasal medium (Gibco #21103-049) containing 2% B27 (Gibco #17504-044), 1% Penicillin/Streptomycin/ Glutamine (Cellgro #30-009-CI) and cultured at 37ºC with 5% CO_2_. After 13 days, cultures were treated with HDAC inhibitors by pin transfer of compound (185 nL per well) using a CyBi-Well vario pinning robot (CyBio Corp., Germany) and subsequently incubated for 24hr at 37^o^C with 5% CO_2_, then fixed in 4% formaldehyde for 10 min. Following two washes with phosphate-buffered saline, cells were permeabilized and blocked with 0.1% Triton X-100, 2% BSA in PBS. Cells were stained with an anti-Ac-H4K12 antibody (Millipore, catalog #04-119) and Alexa488-conjugated secondary antibody (Molecular Probes). Cell nuclei were identified by staining with Hoechst 33342 (Invitrogen, H3570). Cell nuclei and histone acetylation signal intensity were detected and measured using a laser-scanning microcytometer (Acumen eX3, TTP Laptech). Acumen Explorer software was used to identify a threshold of histone acetylation signal intensity such that, without HDAC inhibitor, >99.5 % of cells had intensity levels below the threshold. In the presence of HDAC inhibitors, cells signal intensities above the threshold were scored as “bright green cells” and expressed as a percentage normalized to DMSO controls. EC_50_ values were determined from curve fitting using GraphPad Prism v5 software (GraphPad Software, Inc. La Jolla, CA, USA).

Western Blotting

Protein was extracted by douncing in suspension buffer: 0.1 M NaCl, 0.01 M TrisCl (ph7.6), 0.001 M EDTA (pH 8.0), then immediately diluted with an equal volume of 2x SDS-gel loading buffer: 100 mM TrisCl (ph 6.8), 4% SDS, 20% glycerol, 200 mM DTT. Human Embryonic Kidney (HEK) 293 cells grown to 75% confluence in 96-well plates were treated with 20 μM Cpd-60, SAHA or DMSO as control for 24hr ‘constant’ treatment, followed by media change and 6 hour incubation (‘washout’) as indicated. Media was aspirated and cells collected for Western blotting using 40 μL buffers as detailed above for mouse brain. Solutions for protein extraction were supplemented with 5 mM sodium butyrate to suppress residual HDAC activity. Samples were denatured at 95°C for 5 min, passaged 3x through a 25 gauge needle and centrifuged at 10,000*g* for 10 min at room temperature and protein quantification performed using a BCA protein assay kit #23225 (Pierce). Ten μg of protein extract was used to assess histone acetylation using commercially available antibodies (Millipore) raised against acetylated histone H2B (07-373), H3K9 (07-352), and H4K12 (04-119) with normalization to total levels of histone H3 (07-690) or histone H4 (04-858) using 10-20% Tris Ready Gels and polyvinylidene difluoride membrane, 0.22 μm pore size (both from Bio-Rad). Chemiluminescent detection of immunoreactivity was performed using SuperSignal West Dura Substrate #34076 (Pierce) with subsequent densitometric quantification using Image J software (NIH) and statistical comparison to vehicle-treated controls by two-tailed t-test.

RNA Isolation and Transcriptional Analysis

Frozen mouse brains (*n* = 6/ treatment group) were rapidly dissected at 4°C, isolating medial prefrontal cortex, nucleus accumbens and hippocampus. RNA was isolated using the Qiagen RNeasy Lipid Tissue mini kit, RNA concentration was determined using a NanoDrop spectrophotometer and quality was assessed using Agilent Bioanalyzer PICO chips, with the RNA Integrity Number (RIN) determined at 7.0-8.8 for all samples assayed, indicative of good RNA quality. For microarray experiments on Illumina MouseWG-6 BeadChips, RNA samples were pooled from two biological replicates such that the total number of samples was 27 (3 treatment groups x 3 brain regions x 3 RNA pools representing two mice per pool). All microarray analysis and gene set analysis (GSA) was performed using GeneSpring software (v.11.5.1) with raw signals adjusted using quantile normalization and baseline transformation with the median signal in all samples set to zero. Data were included in subsequent analyses if the normalized signal, thresholded at 0.8, was ‘present’ for 3 of 3 replicates for like treatment and tissue groups. One-way ANOVA tests for treatment effects on transcript expression within each of the three brain regions were run with and without Benjamini-Hochberg correction for false discovery [[4](#_ENREF_4)].Subsequent validation of a set of candidate genes was performed by real-time quantitative PCR (qPCR) using a Roche Lightcycler 480 using 2x SYBR reagent (Roche #04707516001) with quantification of each transcript was performed using ΔΔCt method [[5](#_ENREF_5)] with GAPDH as normalizing gene and statistical comparison to vehicle-treated controls by two-tailed t-test. Primer sequences for qPCR validation were designed using NCBI PRIMER-BLAST (Table S2). For comparison of Cpd-60- or SAHA-induced gene expression changes compared to those reported following lithium treatment, a custom gene list was created using published gene symbols for altered transcripts, exactly as previously reported [[6](#_ENREF_6)], and imported into GeneSpring. GSA analysis was performed using a maximum permutation threshold of 100 and a Benjamini Hochberg FDR threshold of p<0.05 against lists of genes we found altered by Cpd-60 or SAHA treatment in any brain region examined (PFC, NAc or HIP), compared to corresponding vehicle-treated controls (>1.2fold, p<0.05).

Chromatin Immunoprecipitation

Frozen mouse brains treated with Cpd-60 or vehicle (*n* = 4/group), were rapidly dissected at 4°C and nucleus accumbens isolated. Native chromatin (unfixed tissue) was digested with 5 units/mL micrococcal nuclease at 37°C for 7 min followed by hypotonic lysis and debris clearing. From this resulting pool of nucleosomal chromatin (‘Input’), 100uL was removed for ChIP, incubated overnight with 2.5 μg of anti-histone H4K12ac antibody and precipitated using A/G Plus agarose beads (Santa Cruz #SC-2003). Pelleted beads were washed, and protein complexes digested with proteinase K, followed by phenol-chloroform purification of DNA using glycogen to nucleate precipitation at -80°C. DNA was also purified from an equal volume of the ‘Input’ fraction without immunoprecipiation. DNA was applied without further amplification to qPCR using ChIP primers (Table S2) designed using NCBI PRIMER-BLAST at 1 kB and 0.2 kB upstream of the transcription start site (TSS) or 0.5 kB downstream, with amplicons designed at <150 bp to ensure amplification from mono-nucleosomal DNA templates. Quantity of starting DNA was evaluated via ‘cross point’ cycle threshold values via Roche Lightcycler 480 software. The ratio of cycle threshold values from input to ChIP samples (2^(input –ChIP )) were compared from mice treated with Cpd-60 to vehicle.

1. Fass DM, Reis SA, Ghosh B, Hennig KM, Joseph NF, et al. (2013) Crebinostat: A novel cognitive enhancer that inhibits histone deacetylase activity and modulates chromatin-mediated neuroplasticity. Neuropharmacology 64: 81-96.

2. Katragadda M, Magotti P, Sfyroera G, Lambris JD (2006) Hydrophobic effect and hydrogen bonds account for the improved activity of a complement inhibitor, compstatin. J Med Chem 49: 4616-4622.

3. Chou CJ, Herman D, Gottesfeld JM (2008) Pimelic diphenylamide 106 is a slow, tight-binding inhibitor of class I histone deacetylases. J Biol Chem 283: 35402-35409.

4. Benjamini YaHY (1995) Controlling the False Discovery Rate: A Practical and Powerful Approach to Multiple Testing. Journal of the Royal Statistical Society (Methodologial) 57: 289-300.

5. Livak KJ, Schmittgen TD (2001) Analysis of relative gene expression data using real-time quantitative PCR and the 2(-Delta Delta C(T)) Method. Methods 25: 402-408.

6. McQuillin A, Rizig M, Gurling HM (2007) A microarray gene expression study of the molecular pharmacology of lithium carbonate on mouse brain mRNA to understand the neurobiology of mood stabilization and treatment of bipolar affective disorder. Pharmacogenet Genomics 17: 605-617.
